# Synergistic Integration of Mesenchymal Stem Cells and Hydrostatic Pressure in the Expansion and Maintenance of Human Hematopoietic/Progenitor Cells

**DOI:** 10.1155/2018/4527929

**Published:** 2018-02-27

**Authors:** Yun Gyeong Kang, Jee-Yeong Jeong, Tae-Hee Lee, Ho Sup Lee, Jung-Woog Shin

**Affiliations:** ^1^Department of Biomedical Engineering, Inje University, Gimhae, Gyeongsangnam-do, Republic of Korea; ^2^Department of Biochemistry and Cancer Research Institute, Kosin University College of Medicine, Busan, Republic of Korea; ^3^Laboratory for Cancer & Stem Cell Biology, Plant Engineering Institute, Sejong University, Seoul, Republic of Korea; ^4^Department of Internal Medicine, Kosin University College of Medicine, Busan, Republic of Korea; ^5^Department of Health Science and Technology/Cardiovascular and Metabolic Disease Center/Institute of Aged Life Redesign/UHARC, Inje University, Gimhae, Republic of Korea

## Abstract

Ex vivo expansion of hematopoietic stem/progenitor cell (HSPC) has been investigated to improve the clinical outcome of HSPC transplantation. However, ex vivo expansion of HSPCs still faces a major obstacle in that HPSCs tend to differentiate when proliferating. Here, we cocultured HSPCs with mesenchymal stem cells (MSCs) and divided the HSPCs into two fractions according to whether they came into adherent to MSCs or not. Additionally, we used hydrostatic pressure (HP) to mimic the physical conditions *in vivo*. Even nonadherent cells expanded to yield a significantly larger number of total nucleated cells (TNCs), adherent cells maintained the HSPC phenotype (CD34^+^, CD34^+^CD38^−^, and CD133^+^CD38^−^) to a greater extent than nonadherent cells and had superior clonogenic potential. Moreover, applying HP significantly increased the number of TNCs, the frequency of the immature HSPC phenotype, and the clonogenic potential. Furthermore, the genetic markers for the HSPC niche were significantly increased under HP. Our data suggest that the nonadherent fraction is the predominant site of HSPC expansion, whereas the adherent fraction seems to mimic the HSPC niche for immature cells. Moreover, HP has a synergistic effect on expansion and functional maintenance. This first study utilizing HP has a potential of designing clinically applicable expansion systems.

## 1. Introduction

Hematopoietic stem/progenitor cell (HSPC) transplantation is used extensively in the treatment of several blood-related malignant and inherited diseases [1]. HSPCs can be collected from donors by bone marrow aspiration, from peripheral blood after granulocyte colony-stimulating factor mobilization, or from umbilical cord blood (UCB) for autologous and allogeneic transplantation [2]. To date, more than 30,000 HSPC transplantations from UCB have been performed globally. Approximately 10% of all HSPC transplantation is UCB-derived annually, and this number is steadily increasing [3]. Compared with use of other sources, UCB is harvested relatively easily and noninvasively. Moreover, UCB-derived HSPCs enhance long-term immune recovery and decrease graft-versus-host disease. However, a number of clinical limitations are present. For successful clinical outcomes, sufficient numbers of cells (at least 2.5–3 × 10^7^ nucleated cells/kg) are required for graft survival after transplantation; however, the number of HSPCs in a single unit of UCB does not meet this threshold for adult patients [4, 5]. To overcome this limitation, many research groups have focused on ex vivo expansion of HSPCs. Despite many costly studies, clinically optimal expansion strategies for improving the reproducibility and performance of HSPC transplantation remain elusive.

In conventional expansion cultures, HSPCs are cultured in suspension cultures with various combinations of early acting cytokines, growth factors, and other growth-promoting compounds, such as stem cell factor (SCF), FMS-like tyrosine kinase 3 ligand (Flt-3L), interleukin- (IL-) 3, IL-6, thrombopoietin (TPO), erythropoietin (EPO), fibroblast growth factor 1 (FGF-1), and granulocyte colony-stimulating factor (GM-CSF) [5–7]. These conventional culture methods have been found to decrease HSPC self-renewal ability and initial phenotype. To resolve these problems, various strategies such as notch-, StemRegenin 1-, nicotinamide- (NiCord-), and UM171-mediated expansion and prostaglandin e2 (PEG2) coculture methods have been studied [8–10]. In addition, numerous studies have focused on mimicking the in vivo microenvironment of HSPCs—referred to as the HSPC niche—which regulates HSPC fates such as self-renewal, quiescence, differentiation, homing, and mobilization.

HSPCs reside in a complex stem cell niche. There are at least 3 different types of marrow niches such as endosteal, reticular, and vascular niche that have biological and physiological effects on HSPC. One of the cells composing these HSPC niche is mesenchymal stem cells (MSCs) which control important functions of HSPCs through direct cell-to-cell contacts, the formation of an extracellular matrix network, and secretion or suppression of endogenous cytokines and factor such as C-X-C motif chemokine 12 (CXCL12, also known as stromal cell-derived factor 1, SDF1), SCF, angiopoietin 1 (ANGPT1), vascular cell adhesion molecule 1 (VCAM1), and TPO [8–10]. Because of the various roles of MSCs, MSCs have been extensively used in coculture system for ex vivo expansion to mimic the natural marrow environment. Unfortunately, expanded HSPCs lack long-term engraftment potential [11, 12], and optimal coculturing techniques have not been developed. Moreover, as recent research has emphasized the importance of a three-dimensional architecture mimicking the physiological condition of the HSPC niche, there is an increasing trend to more closely examine the interaction between HSPCs and MSCs [13–15]. In other words, although studies have traditionally considered HSPCs in a coculture system as a single population, it is necessary to separate and analyze the localization of the HSPC relative to the MSC layers to gain insight into the constitution and function of the HSPC-MSC microenvironment *in vitro.*

Bioreactor-based systems have been employed to mimic the functional HSPC niche [16–18]. Such systems facilitate HSPC culture under dynamic conditions by mimicking the mechanical factors experienced by HSPCs *in vivo*. Additionally, such systems might facilitate mass transportation and homogenous status in cultures. Several types of bioreactors, including the fixed-bed bioreactor [19], stirred suspension bioreactor [20], rotating wall vessel (RWV) [21], and perfusion chamber [22], have been designed to maintain cell function during expansion by modulating various factors. Most studies using bioreactors for HSPC expansion have fallen short of reproducibility in terms of expansion efficiency, and no further studies using bioreactors have been reported. The present study introduces a bioreactor that employs hydrostatic pressure (HP) to mimic the mechanical environment in bone marrow. To our knowledge, the HP present in the intramedullary structure of bones has not been previously evaluated. The systemic blood pressure in animals has been reported to be in the range of 110–140 mmHg, whereas the normal intramedullary pressure is, on average, 30 mmHg [23].

To address some of these unresolved problems, we cocultured HSPCs with MSCs, which are known as one of the cells constituting HSPC niche. After 4 days, expanded HSPCs were analyzed by separation according to their localization relative to the MSC layers. We assessed whether the outcomes of the coculture system were caused by adherence to MSCs, by the soluble factors secreted by the cells in culture, or by other variables. Furthermore, we systematically evaluated combinations of HP (with or without MSCs) to determine the most favorable culture conditions for effective expansion. This study provides insight into the optimal design of the artificially engineered niche for the ex vivo expansion and functional maintenance of HSPCs.

## 2. Materials and Methods

### 2.1. Preparation of MSCs

Human bone marrow-derived MSCs were purchased from Lonza (Walkersville, MD, USA) and cultured in MSC Growth Medium BulletKit (Lonza). Cells were incubated at 37°C in a humidified incubator containing 5% CO_2_, fed twice per week, and used at passage number 4 for all experiments.

### 2.2. CD34^+^ HSPC Culture

Frozen human UCB CD34^+^ HSPCs were purchased from StemCell Technologies (Vancouver, BC, Canada) and used as provided (passage number 1). The CD34^+^ cell purity in the HSPCs was determined to be 98% by flow cytometry, and the viability was determined to be >97% by trypan blue staining.

CD34^+^ HSPCs were cultured in StemSpan SFEM II expansion medium (StemCell Technologies) with 100 ng/ml SCF (ProSpec, Rehovot, Israel), 100 ng/ml Flt-3L (ProSpec), 50 ng/ml TPO (ProSpec), 20 ng/ml IL-3 (ProSpec), and 20 ng/ml IL-6 (ProSpec). The initial inoculum and maintenance densities were 5 × 10^4^ and 1 × 10^6^ cells/ml, respectively. Culture media were exchanged with fresh media at one-half volume of the initial media every 3 days, and the cultures were maintained at 37°C in a humidified atmosphere containing 5% CO_2_.

### 2.3. Coculture of HSPCs with MSCs

Before establishing cocultures, human MSCs were seeded in six-well plates (SPL Life Sciences, Pocheon-si, Gyeonggi-do, Korea) at a density of 1.5 × 10^5^ cells/well in MSC growth medium. The day after seeding the MSCs, HSPCs were suspended in HSPC expansion media, as described previously. HSPC suspensions were plated at an inoculum density of 5 × 10^3^ cells/ml on confluent MSC at 37°C in 5% CO_2_. Half the medium was changed every 3 days for cytokine replenishment.

To confirm the effects of direct cellular interaction between HSPCs and MSCs, nonadherent HSPCs were harvested and carefully isolated as in the previous study [24]. Briefly, the supernatant of the coculture was harvested and centrifuged to collect nonadherent fraction. The MSC layer was washed twice using phosphate-buffered saline (PBS; Sigma-Aldrich, St. Louis, MO, USA) with 1% (vol/vol) bovine serum albumin (BSA; MP Biomedicals, Solon, OH, USA) to remove the remaining nonadherent cells. After removing all the nonadherent fractions that could be removed through a gentle washing process, the cells remaining on the MSC layer were harvested by further intensive washing procedures with aforementioned solution. After washing, the remaining HSPCs, which were more strongly attached to the MSC layer, were collected by trypsin treatment and all procedures were carried out using a bright-filed microscope (Olympus, Tokyo, Japan). To rule out nonspecific effects mediated by enzyme digestion, nonadherent HSPCs were also incubated with trypsin for 5 min.

### 2.4. Mechanical Stimulation

A novel bioreactor system was used to apply HP to cells (ACBC-100; AnyCasting, Gimhae-si, Korea). Based on our previous work [25], the HP treatment regime applied to all groups involved 2 and 15 min for stimulating and resting, respectively. HP was applied 4 h/day for 2 days at a magnitude of 20 kPa, starting 2 days after coculturing. All experimental data were collected after 1, 3, and 4 days, allowing investigation of the ongoing effects of the mechanical stimuli, even after cessation of HP treatment. The investigations involved six groups: H, H_HP, H_M_NA, H_M_NA_HP, H_M_A, and H_M_A_HP. “H” and “M” indicate HSPCs and MSCs, respectively. “NA” and “A” indicate the localizations of HSPCs. “NA” refers to HPSCs that were nonadherent cells to MSCs. “A” indicates HPSCs that were adherent cells to MSCs. “HP” indicates a group that was subjected to mechanical stimulation.

### 2.5. Hematopoietic Cell Counts

CD34^+^ HSPCs were seeded at an initial density of 1000 cells/well in 96-well plates (SPL Life Sciences) under the culture conditions indicated in the text. Cell counting was performed after 0, 1, 3, and 4 days with a Countess II FL Automated Cell Counter (Thermo Fisher Scientific Inc., Waltham, MA, USA) within a diameter range of 7 to 20 *μ*m. The fold increase in total nucleated cell (TNC) count was calculated by dividing the number of cells (at each day) by the initial number of cells (on day 0).

### 2.6. Scanning Electron Microscopy (SEM) of HSPCs on MSC Layers

To determine the morphology of the HSPCs on MSC layers, suspended HSPCs were collected separately, and the attached HSPCs were observed. Adherent HSPCs to MSCs were rinsed gently with PBS (Thermo Fisher Scientific Inc.), fixed with 2.5% (vol/vol) glutaraldehyde (Sigma-Aldrich, St. Louis, MO, USA) in PBS for 30 min at room temperature, and post-fixed with 1% osmium tetraoxide (Sigma-Aldrich) for another 15 min at room temperature. Samples were then dehydrated using a graded series of ethanol (50%, 70%, 90%, 95%, and 100%), each for 5 min, and the samples were dried in an aseptic environment for 24 h. The samples were mounted onto aluminum stubs and gold sputter-coated before observation using scanning electron microscopy (SEM; Hitachi Science Systems Ltd., Tokyo, Japan).

### 2.7. Flow Cytometry

Expanded cell suspensions were washed with PBS containing 0.2% (vol/vol) BSA and 0.09% sodium azide (Sigma-Aldrich) as a staining buffer. After blocking the nonspecific binding of antibodies using 5% BSA in staining buffer, cells were stained for 30 min at 4°C with fluorochrome-labeled mouse or rat anti-human monoclonal antibodies as follows: CD34 R-phycoerythrin (PE; BD Biosciences, San Jose, CA, USA), CD38 fluorescein isothiocyanate (FITC; BD Biosciences), and CD133 APC (BD Biosciences). Cells stained with isotype-matched antibodies were used as controls. Cells were washed carefully with the staining buffer and suspended in exactly 500 *μ*l 1% paraformaldehyde (USB, Fremont, CA, USA).

Acquisition was performed on the BD FACSCanto II flow cytometer (BD Biosciences), and the analyses were performed using FACSDiva version 6.1.3 software (BD Biosciences). A minimum of 10,000 events were collected for each sample.

### 2.8. Colony-Forming Cell Assay

Colony-forming cell (CFC) assays for granulocyte and macrophage (CFU-GM), erythroid (burst-forming unit-erythroid (BFU-E)), and multilineage (CFU-granulocyte, erythrocyte, monocyte, and megakaryocyte (CFU-GEMM)) progenitors were performed. Expanded TNCs from each group were plated in triplicate at a density of 10^3^ cells in a 35 mm dish in semisolid methylcellulose medium (MethoCult GF H4434; StemCell Technologies). After 14 days of culture in a humidified environment at 37°C and 5% CO_2_, the colonies consisting of 50 or more cells were observed under a stereomicroscope (SMZ745T; Nikon, Tokyo, Japan).

### 2.9. Long-Term Culture-Initiating Cell Assay

A modified LTC-IC assay was performed as previously described [26]. Briefly, mitomycin c- (MMC-; Sigma-Aldrich) treated (20 *μ*g/ml, for 3 h) mouse bone marrow stromal cells (M2-10B4; Korean Cell Line Bank, Seoul, South Korea) were seeded at 3 × 10^5^ cells/well in a 35 mm culture dish as a feeder layer the day before the assay. In duplicate, 5 × 10^5^ cells from expanded cells of each group were seeded in 35 mm culture dishes with the MMC-treated MSCs as a feeder layer in 2.5 ml MyeloCult H5100 (StemCell Technologies) supplemented with 10^−6^ M hydrocortisone. The plates were incubated at 37°C, 5% CO_2_ with weekly half-medium exchanges. After 6 weeks of culture, the cells were harvested and transferred (5 × 10^4^ cells, in triplicate) to methylcellulose medium with recombinant cytokines (MethoCult H4435; StemCell Technologies) to score secondary CFCs. After 14 days of culture, colonies with >50 cells were counted to assess LTC-IC activities, and the frequency of LTC-IC was calculated according to the manufacturer's instructions (StemCell Technologies).

### 2.10. RNA Extraction and Quantitative Real-Time Polymerase Chain Reaction (qRT-PCR)

RNA was purified using the RNeasy Mini Kit (Qiagen, Hilden, Germany) according to the manufacturer's instructions [27]. RNA was preincubated with DNase I (Invitrogen), and reverse transcription was performed with a high-capacity RNA to cDNA kit (Applied Biosystems, Foster, CA, USA) as per the manufacturer's instructions [27]. Quantitative real-time (qRT) polymerase chain reaction (PCR) was performed on cDNA using Power SYBR® Green PCR Master Mix (Applied Biosystems). Data analysis was performed using the StepOne Real-Time PCR System (Applied Biosystems) and QuantStudio 3 Real-Time PCR System (Applied Biosystems) according to the 2^−ΔΔCt^ method. qRT-PCR analyses were performed three times for each sample. Product size and primer sequences used were as per Supplementary Table S1.

### 2.11. Gene Expression Profiling Using a Quantitative PCR Array

To analyze gene expression in expanded HSPCs after coculture with MSCs and HP treatment, RNA was purified and converted to cDNA as previously described. Several sets of primers (Supplementary Table S2) were designed by Bioneer Corporation (Daejeon, South Korea). Gene amplification of each cDNA sample was performed using Power SYBR Green Master Mix and the QuantStudio 3 Real-Time PCR System (Applied Biosystems) according to the 2^−ΔΔCt^ method. The entire system and all experiments involved were performed according to the minimum information for publication of Quantitative Real-Time PCR Experiments (MIQE) guidelines. Analyses were performed in triplicate. Data analysis was based on the relative quantification software of Thermo Fisher Cloud, provided by Thermo Fisher Scientific incorporated. Expression levels were normalized to those of reference genes, including hydroxymethylbilane synthase (HMBS), beta-2-microglobulin (B2M), hypoxanthine phosphoribosyltransferase 1 (HPRT1), glyceraldehyde-3-phosphate dehydrogenase (GAPDH), and phosphoglycerate kinase 1 (PGK1).

### 2.12. Statistical Analyses

The significance of differences was assessed by one-way analysis of variance (ANOVA) using SPSS (PASW Statistics 22; SPSS Inc., USA). When ANOVA indicated a significant difference among groups, the difference was evaluated using the least-significant difference (LSD). All data are presented as the means ± standard deviation (SD), with the significance level set at *p* < 0.05.

## 3. Results

### 3.1. Total Nucleated Cell Expansion

**Figure 1:**
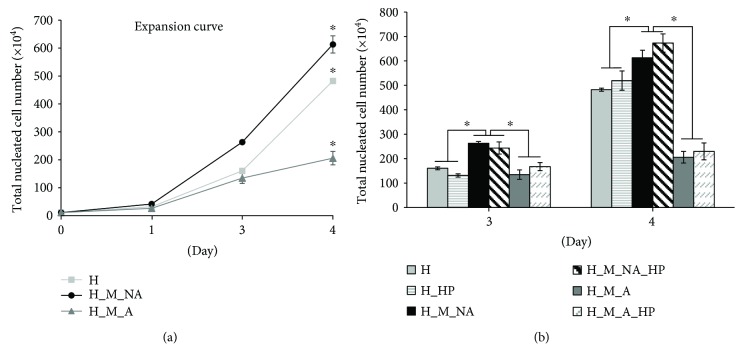
Total nucleated cell (TNC) expansion. (a) Expansion curves of TNCs for 4 days in culture according to coculture with mesenchymal stem cells (MSCs) and adherence. (b) Changes in the number of TNCs after applying hydrostatic pressure (HP) on days 3 and 4 (*n* = 5, ^∗^ *p* < 0.05).

To determine the influence of cellular localization on TNC expansion, we counted the number of cells in each of two groups: those that were in adherence to MSCs (A group) and those that were not (NA group). Throughout 4 days of coculture, there was a significant difference among the three groups: the H_M_NA group and H group (control) increased similarly and much faster than the H_M_A group (Figure 1(a)).

**Figure 2:**
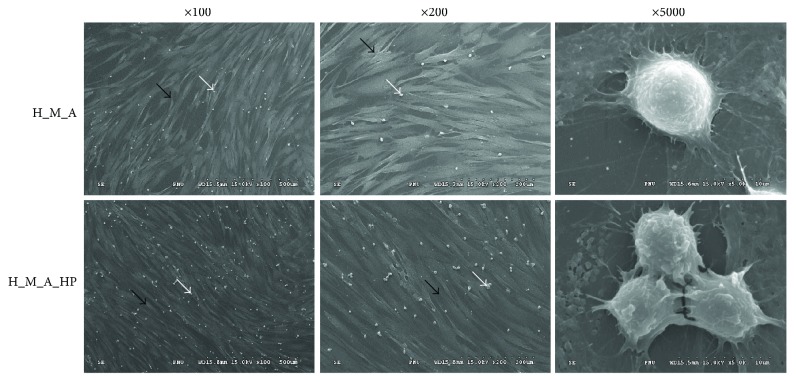
Scanning electron micrograph (SEM) images of hematopoietic stem/progenitor cells (HSPCs; white arrows) cultured directly over mesenchymal stem cell (MSC) feeder layers (black arrows). Day 4 cocultures are presented using different magnifications (×100, ×200, and ×5000) showing well-established cell-cell interactions between HSPCs and MSCs.

We assessed the number of TNCs with and without the application of HP on day 3. We observed no significant difference in TNC expansion between the HP-treated and non-HP-treated groups. All groups with HP (H_HP, H_M_NA_HP, and H_M_A_HP groups) showed a tendency to have increased numbers of TNCs compared to the group that was not subject to HP on day 4. Similar results were obtained in SEM images (Figure 2), which confirmed the morphology of the HSPCs that were in adherence to the MSC layer at day 4. When HP was applied, a larger number of “adherent” HSPCs could be identified, and HSPCs clustered together.

As nonadherent cells (NA group) and adherent cells (A group) were obtained from the same wells, we can conclude that HSPC expansion was clearly enhanced by coculture with MSCs rather than by cytokine and growth factors alone (H: 44.28 ± 0.57-fold; H_HP: 47.69 ± 3.62-fold; H_M: 75.18 ± 4.6-fold; H_M_HP: 82.9 ± 5.8-fold).

### 3.2. Surface Marker Expression of Expanded Cells

To investigate the impact of HP and adherence to MSCs on HSPC differentiation, HSPC phenotypes were determined by flow cytometry. CD34 is a typical HSPC marker [28], and CD34^+^CD38^−^ cells and CD133^+^CD38^−^ cells are usually considered to be a more primitive HSPC population [29].

**Figure 3:**
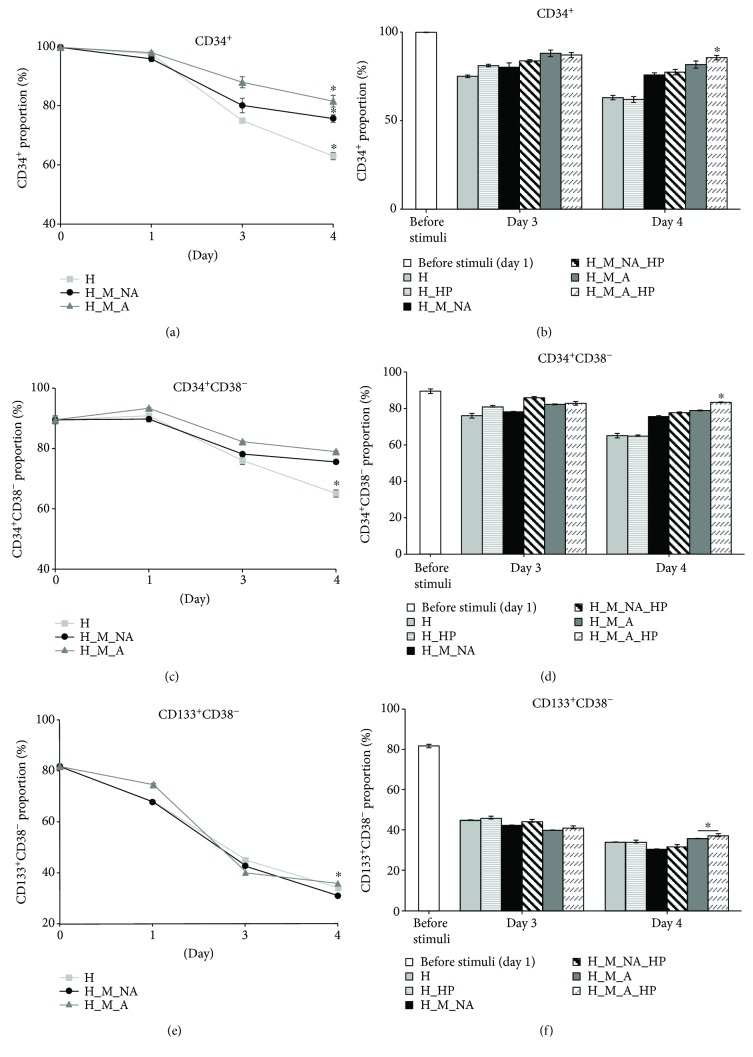
Flow cytometry analysis of expanded cells under different culture conditions. Proportion of cells expressing the typical HSPC marker (a) CD34^+^ and primitive HSPC markers (c) CD34^+^CD38^−^ and (e) CD133^+^CD38^−^ according to coculture with MSCs and adherence. Proportion of cells expressing (b) CD34^+^, (d) CD34^+^CD38^−^, and (f) CD133^+^CD38^−^ after applying hydrostatic pressure (HP) on days 3 and 4, (*n* = 3, ^∗^ *p* < 0.05).

Based on surface marker expression after 4 days without considering applying HP in culture, HSPCs cocultured with MSCs (i.e., the H_M_NA and H_M_A groups) maintained their phenotype (CD34^+^ and CD34^+^CD38^−^; Figures 3(a) and 3(c)) longer than HSPCs cultured alone (H group). The fraction of CD133^+^CD38^−^ cells followed a similar pattern after 3 days (Figure 3(e)).

A higher proportion of cells in the HP-treated groups (i.e., the H_HP, H_M_NA_HP, and H_M_A_HP groups) than in the non-HP-treated groups (H, H_M_NA and H_M_A) maintained the HSPC phenotype (CD34^+^, CD34^+^CD38^−^, and CD133^+^CD38^−^) (Figures 3(b), 3(d), and 3(f)). However, HP did not appear to affect the maintenance of the HSPC phenotype when HSPCs were cultured alone (i.e., in the H and H_HP groups). Notably, expanded cells in the H_M_A_HP group retained their phenotype (CD34^+^, CD34^+^CD38^−^, and CD133^+^CD38^−^) on days 3 and 4, whereas those in the other groups differentiated significantly compared to previous days (*p* < 0.05).

### 3.3. Clonogenic Potential of Expanded Cells

**Figure 4:**
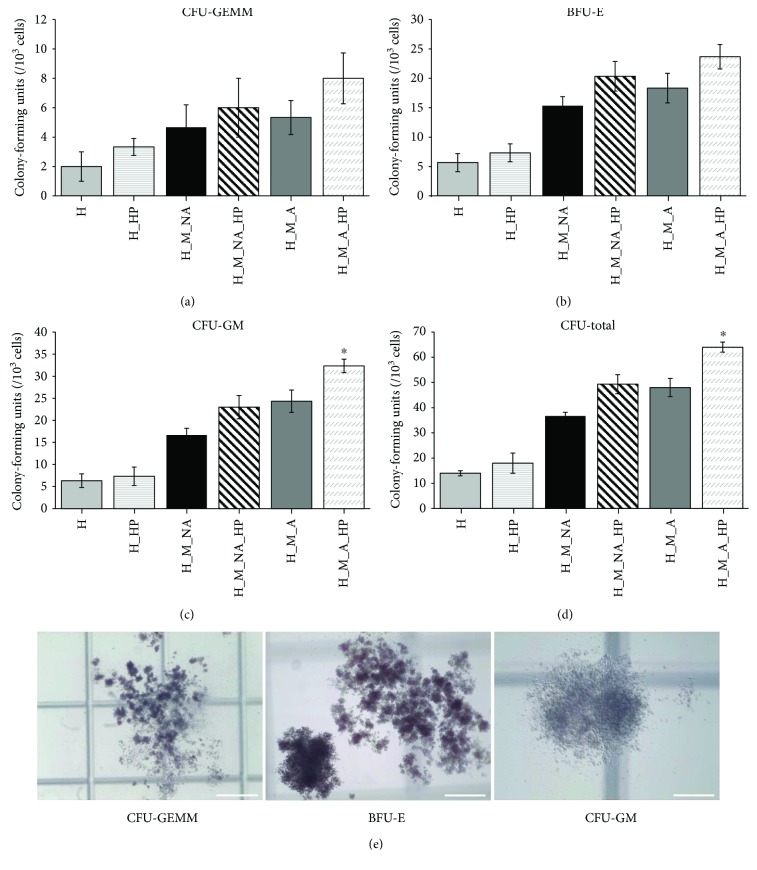
Clonogenic potential of expanded cells. (a) CFU-GEMM, (b) BFU-E, (c) CFU-GM, and (d) total CFU counts after 14 days in culture, using cells cultured for 4 days under various conditions (*n* = 3, ^∗^ *p* < 0.05). (e) Morphological evaluation of CFUs in the H_M_A_HP group after 4 days in culture. Images are representative of three data sets (CFU-GEMM, bar = 200 *μ*m; BFU-E and CFU-GM, bar = 100 *μ*m). CFU-GEMM: colony-forming units-granulocyte, erythrocyte, monocyte, and megakaryocyte; BFU-E: burst-forming unit-erythroid; CFU-GM: colony-forming unit-granulocyte and macrophage.

**Figure 5:**
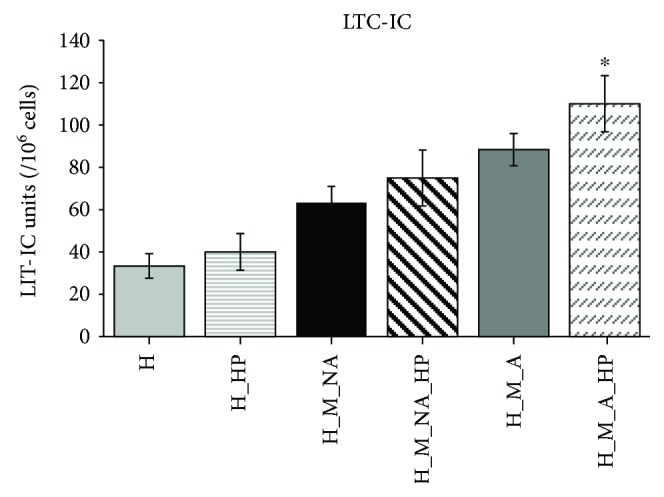
Secondary clonogenic potential of expanded cells. Cells cultured for 4 days under different conditions were cultured for 6 additional weeks in long-term culture-initiating cell (LTC-IC) assays, and colony-forming cell (CFC) assays were then performed (*n* = 3, ^∗^ *p* < 0.05).

To determine whether the expanded HSPCs were functional, we investigated the clonogenic capacity of expanded HSPCs using CFC (Figure 4) and LTC-IC assays (Figure 5) *in vitro*. Regardless of HP application, the cocultured groups (H_M_NA, H_M_NA_HP, H_M_A, and H_M_A_HP) produced significantly more BFU-E and CFU-GM colonies compared with the HSPC single-culture groups (H and H_HP). Furthermore, more total colonies were produced by HSPCs cocultured with MSCs than by HPSCs cultured alone. When colony formation was compared among cocultured groups, the groups containing cells that were adherent to MSCs (H_M_A and H_M_A_HP) formed significantly more BFU-E and CFU-GM colonies compared with the groups containing cells that did not come into adherent to MSCs (H_M_NA and H_M_NA_HP). In particular, the H_M_A_HP group formed more colonies than other groups. However, there were no significant differences among groups in terms of the number of CFU-GEMM colonies.  Figure 4(e) shows representative images of CFU-GEMM, BFU-E, and CFU-GM colonies generated by the H_M_A_HP group.

Similar results were also observed using the LTC-IC assay. The cocultured groups (H_M_NA, H_M_NA_HP, H_M_A, and H_M_A_HP) formed more secondary colonies than the single culture group (H and H_HP), and HP application (in the H_HP, H_M_NA_HP, and H_M_A_HP groups) resulted in more secondary colonies compared with the non-HP-treated groups (H, H_M_NA and H_M_A). In other words, HSPCs that adhered to MSCs and were subjected to HP (H_M_A_HP) produced significantly more secondary colonies (33.33 ± 5.77 versus 40 ± 8.66 versus 63.33 ± 7.63 versus 75 ± 13.22 versus 88.33 ± 7.63 versus 110 ± 13.22; H versus H_HP versus H_M_NA versus H_M_NA_HP versus H_M_A versus H_M_A_HP; *n* = 3), indicating that these HSPCs had a greater repopulating capacity.

### 3.4. Expression of HSPC Niche Markers in Cocultured MSCs

**Figure 6:**
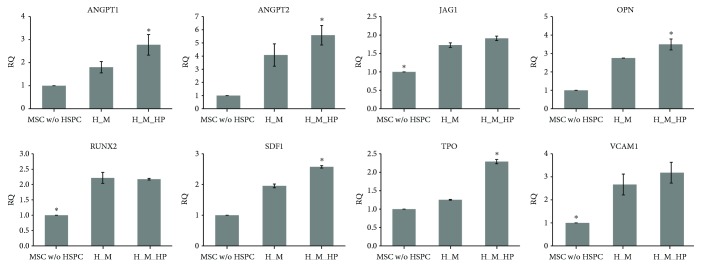
Gene expression of HSPC niche markers. The relative gene expression of HSPC niche markers (ANGPT1, ANGPT2, JAG1, OPN, RUNX2, SDF1, TPO, and VCAM1) was confirmed by harvesting MSCs cocultured with HSPCs with or without the application of HP. Additionally, single cultured MSCs were used (MSCs without HSPC) as a control (*n* = 5, ^∗^ *p* < 0.05). ANGPT1: angiopoietin 1; ANGPT2: angiopoietin 2; JAG1: jagged 1; OPN: osteopontin; RUNX2: runt-related transcription factor 2; SDF1: stromal cell-derived factor 1; TPO: thrombopoietin; VCAM1: vascular cell adhesion molecule 1.

To identify the mechanisms underpinning the increased expansion observed among HSPCs cocultured with MSCs under HP, we performed qRT-PCR to investigate the relative expression of several HSPC niche markers: ANGPT1, angiopoietin 2 (ANGPT2), jagged 1 (JAG1), osteopontin (OPN), runt-related transcription factor 2 (RUNX2), SDF1, TPO, and VCAM1. These markers are known to be essential for HSPC survival *in vivo* and to be expressed by niche support cells [30–32]. After 4 days of culture, we harvested MSCs cultured with or without HSPCs/HP and assessed their relative gene expression (Figure 6). We used MSCs cultured without HSPCs as a control.

MSCs cocultured with HSPCs expressed significantly higher levels of all markers compared with MSCs cultured alone. In particular, cocultured MSCs that were subjected to HP (H_M_HP group) expressed significantly higher levels of most markers (ANGPT1, ANGPT2, JAG1, OPN, SDF1, and TPO) compared with cocultured MSCs that were not subjected to HP (H_M group).

### 3.5. Identification of Expanded Cells according to HSPC- and Hematopoiesis-Related mRNA Expression

**Figure 7:**
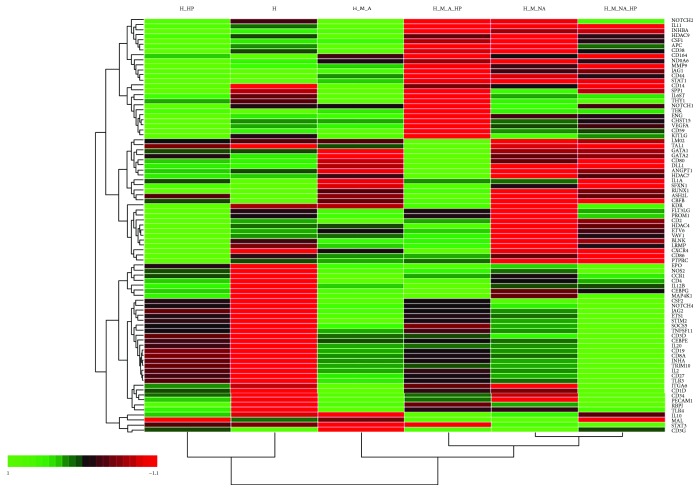
Representative heatmap. Quantitative polymerase chain reaction (qPCR) arrays identified genes associated with blood-related surface/lineage markers, cytokine and growth factors, cell cycle regulators, blood cell activation, blood-related differentiation, hematopoiesis regulators, and signaling molecules. The overall gene expression pattern of each group was analyzed using hierarchical clustering. Each sample is arranged on the horizontal axis, and genes are arranged on the vertical axis. The order of arrangement is based on the similarity of gene expression patterns (*n* = 3).

HSPCs cultured under different conditions showed proliferative, phenotypic, and functional differences. To elucidate the molecular basis for these differences, we compared the gene expression profiles among fractions using qRT-PCR arrays.  Figure 7 shows the overall gene expression patterns analyzed using hierarchical clustering. In particular, we focused on the expression of genes associated with blood-related surface/lineage markers, cytokine and growth factors, cell cycle regulators, blood cell activation, blood-related differentiation, hematopoiesis regulators, and signaling molecules.

The results confirmed that the pattern of gene expression in expanded HSPCs depended on coculture conditions, adherence to MSCs, and exposure to HP. Cocultured HSPCs and HSPCs cultured alone formed separate clusters. Additionally, hierarchical clustering showed clear differences between the adherent and nonadherent fractions of HSPCs cocultured with MSCs. In particular, the H, H_HP, and H_M_A groups showed generally higher expression of genes encoding cell surface and cell lineage markers (e.g., CD44, THY1, TEK, ENG, CHST15, CD 59, and KITLG) and blood cell differentiation markers (e.g., NOTCH2, IL-11, INHBA, CSF1, and NCOA6) compared with other groups. Interestingly, transcription factors and regulatory genes such as TAL1, GATA1, GATA2, RUNX1, ASH2L, and CBFB were expressed at lower levels in the H_M_NA and H_M_NA_HP groups. On the other hand, the expression of cytokine- and growth factor-related genes (CSF2, TNFSF11, INHA, IL-20, TLR3, etc.), of blood cell differentiation-related genes (e.g., MAP4K1, CD3G, MAL, TRL4, and IL-20), and of signal transduction-related genes (e.g., NOTCH4, SOCS5, STAT3, and CD3G) were higher in HSPCs cultured with MSCs than in HSPCs cultured alone.

## 4. Discussion

Many studies have indicated that HSPCs can be expanded in serum-free culture supplemented with cytokines such as SCF, TPO, and IL-3 [6–8]. However, these culture conditions result in robust expansion accompanied by concomitant differentiation and rapid loss of stemness, leading to loss of HSPC function. Over the past few years, it has been shown that MSCs of the endosteal niche can be used to control and support the long-term repopulation and self-renewal of HSPCs [11–15]. Nevertheless, the mechanisms involved in HSPC regulation remain undefined owing to the limited availability of optimal ex vivo culture models that mimic the HSPC niche. Recently, multiple studies have underscored the need for mimicking the HSPC niche by coculturing HSPCs with other niche cells using various methods [33–35]. However, the conditions for expanding immature hematopoietic cells in an undifferentiated state remain elusive. The optimal combination and quantity of cytokines, time in culture, initial cell density, enrichment of CD34^+^ cells, and other factors have not yet been fully determined. Indeed, a simple cocktail of cytokines may not be sufficient to provide the essential cues for HPSC maintenance and expansion.

In the present study, we first focused on interactions between HSPCs and MSCs to investigate the existence of adherent-dependent effects on the proliferative, phenotypic, and clonogenic potential of HSPCs. Unfortunately, previous studies focusing on HSPCs cocultured with other stromal cells analyzed expanded HSPCs as a single population: cells floating in the medium were not distinguished from cells attached to the feeder layer. In contrast, we separated HSPCs that were nonadherent from those that were adherent to the surface of the MSC layer. As expected, the expanded TNC count for nonadherent cells was significantly higher than that of adherent cells (Figure 1(a)). However, the adherent cells maintained the HSPC phenotype (CD34^+^, CD34^+^CD38^−^, and CD133^+^CD38^−^) to a greater degree than the nonadherent cells over the course of 4 days (Figures 3(a), 3(c), and 3(e)). The adherent cells also had superior clonogenic potential as assessed by CFC and LTC-IC assays (Figures 4 and 5).

These results clearly indicate that HSPC expansion is enhanced by cellular interactions with MSCs rather than by cytokine and growth factors alone. Moreover, we demonstrated that adherent cells contained more relatively undifferentiated cells and retained greater clonogenic capacity. These results are consistent with several previous studies showing the importance of adherence to stromal cells for HSPC expansion [24, 36]. However, few studies have performed a direct comparison among adherent HSPCs, nonadherent HSPCs, and HSPCs cultured alone, focusing on the time course of HSPC coculture.

We also used a bioreactor to provide hydrostatic pressure to reproduce the physical conditions of the HSPC niche. Various types of bioreactor have already been evaluated for ex vivo HSPC expansion [16–22]. For example, the fixed-bed bioreactor developed by Meissner et al. [19] achieved moderate (sevenfold) expansions of the CFU-GM population, but the authors did not evaluate the engraftment potential of expanded cells. Previously evaluated bioreactors still face issues such as lack of reproducibility, lack of clonogenic potential, and low engraftment capacity for expanded HSPCs. Liu et al. [21] reported that the RWV bioreactor achieved a 435 ± 87.6-fold expansion of total cells, a 32.7 ± 15.6-fold expansion of CD34^+^ cells, and a 21.7 ± 4.9-fold expansion of CFU-GM; however, the engraftment potential of the expanded cells was not evaluated. To overcome these limitations, we utilized a bioreactor that supplies HP, a key mechanical element in bone marrow [23], to promote MSC differentiation into specific lineages and proliferation for tissue engineering applications [37–39].

We found that expanded cells from groups subjected to HP tended to have higher numbers of TNCs than did those from groups that were not subjected to HP (H versus H_HP, H_M_NA versus H_M_NA_HP, and H_M_A versus H_M_A_HP) (Figures 1(b) and 2). Interestingly, when HP was applied, the HSPC phenotype frequency (CD34^+^, CD34^+^CD38^−^, and CD133^+^CD38^−^) (Figures 3(b), 3(d), and 3(f)) and clonogenic potential (Figures 4 and 5) were significantly higher than when HP was not applied. In other words, expanded adherent HSPCs exposed to HP (H_M_NA_HP group) retained the HSPC phenotype (CD34^+^, CD34^+^CD38^−^, and CD133^+^CD38^−^) for the longest time and had the highest clonogenic potential as assessed by CFC and LTC-IC assays. However, when HSPCs were cultured without MSCs, HP application had little effect on the maintenance of the HSPC phenotype or the clonogenic potential of expanded cells. Therefore, HP and MSC coculture had synergistic effects.

To identify the cause of these synergistic effects, we harvested cocultured MSCs under different culture conditions and assessed gene expression for HSPC niche markers (ANGPT1, ANGPT2, JAG1, OPN, RUNX2, SDF1, TPO, and VCAM1) that are essential for HSPC survival *in vivo* [8–10]. As expected, we found that the expression levels of all markers were significantly higher in MSCs cocultured with HSPCs (H_M and H_M_HP) compared with those with MSC alone (MSC without HSPC) (Figure 6). These results are consistent with previous studies focusing on HSPCs cocultured with stromal cells [40, 41]. Furthermore, the present study revealed that the expression of HSPC niche markers differed significantly depending on the presence or absence of HP. This result supports the notion that mechanical stimuli can modulate the expression of specific genes [42, 43]. Moreover, HP may affect autocrine/paracrine signaling by MSCs to maintain and expand HSPCs.

We performed extensive gene expression profiling under different culture conditions (Figure 7). Importantly, we found that that coculture and HP treatment can modulate the expression of genes associated with blood-related surface/lineage markers, cytokine and growth factors, cell cycle regulators, blood cell activation, blood-related differentiation, hematopoiesis regulators, and signaling molecules. However, we did not determine the precise underlying mechanisms for these effects. Therefore, future research should address the biological relevance of these upregulated and downregulated genes. Also, our analysis of adherent and nonadherent cells collected from cocultures leaves much room for further investigation. Our analysis assumed that two types of cells, adherent and nonadherent, remained in that state once they are attached or not attached, respectively, to the MSCs. There might be constant exchanges of the cells between two fractions of the adherent and nonadherent cells, which would affect gene expression profiles and other responses.

## 5. Conclusions

Our study demonstrated that the direct contact between HPSCs and MSCs enhances the maintenance of an immature HSPC phenotype and function. Additionally, we showed that HP application significantly influences the outcome of HSPC expansion and the maintenance of stemness. We reached the following conclusions: (1) Coculture of HSPCs with MSCs is crucial for the efficient expansion of TNC, as well as HSPCs. (2) When HSPCs cocultured with MSCs are harvested and analyzed according to their adherence or nonadherence to MSCs, the adherent fraction retains a more immature HSPC phenotype and has greater clonogenic potential than the nonadherent fraction. (3) HP (20 kPa) is effective for HSPC expansion and phenotype maintenance, and HP has synergistic effects with cell-cell interactions. Ours is the first study to apply HP for the expansion of HSPCs. These results are expected to have an important impact on the development of efficient clinical scale expansion systems. The HP system uses a multidirectional expansion strategy suitable for clinical use, alone or in combination with other expansion protocols, including coculture. It also provides a platform with which to examine the dynamic properties of the *in vitro* HSPC culture system.

Xenotransplantation and secreted factor analyses should be performed to investigate the long-term maintenance of the HSPC phenotype. Additionally, various magnitudes of HP should be evaluated to determine the optimal regime for efficient HSPC expansion.

## Supplementary Materials

Supplementary Materials Supplementary Table S1: primers used for quantitative real-time polymerase chain reaction. Supplementary Table S2: genes evaluated in the quantitative PCR array.
