# Supplementary material for: Synergistic Integration of Mesenchymal Stem Cells and Hydrostatic Pressure in the Expansion and Maintenance of Human Hematopoietic/Progenitor Cells
Source: Stem Cells Int. 2018 Feb 27;2018:4527929. doi: 10.1155/2018/4527929 (PMC5848107; doi:10.1155/2018/4527929)
Supplement: Supplementary Materials — Supplementary Table S1: primers used for quantitative real-time polymerase chain reaction. Supplementary Table S2: genes evaluated in the quantitative PCR array. [file 4527929.f1.pdf]

Supplementary Tables

Supplementary Table S1. Primers used for quantitative real-time polymerase chain reaction

Marker		Sequence (5'→3')	Accession No.
Angiopoietin 1, ANGPT1	A	CCCTCCGGTGAATATTGGCTGG	NM_001146.3
	AS	CTCGGTTCCCTTCCCAGTCCATTA	
Angiopoietin 2, ANGPT2	A	AAATAAGCAGCATCAGCCAACCAGG	NM_001147.2
	AS	CACATGCATCAAACCACCAGCCTC	
Jagged 1, JAG1	A	CATCGGGGGCAACACCTTCAA	NM_000214.2
	AS	AATCCCACGCCTCCACAAGCA	
Osteopontin, OPN	A	GGAAAGCGAGGAGTTGAATGGTGC	NM_000582.2
	AS	GGACTGCTTGTGGCTGTGGGTTTC	
Runt-related transcription factor 2, RUNX2	A	TGAGATTTGTGGGCCGGAGTGG	NM_001024630.3
	AS	TCTGTGCCTTCTGGGTTCCCGA	
Stromal cell-derived factor 1, SDF1	A	CCCGGATTGCTGCTCAGATTGG	NM_199168.3
	AS	GGCTCAGGGTCAGCGTGGAATT	
Thrombopoietin, TPO	A	TGATGGCAGCACGGGGACAA	NM_000460.3
	AS	AGGCTCTGCAGGGCCCCAAG	
Vascular cell adhesion molecule 1, VCAM1	A	GACCCCGGATTGCTGCTCAGATT	NM_001078.3
	AS	CTCAGGGTCAGCGTGGAATTGG	
Beta-actin, B-actin	A	GAGCGGGAAATCGTGCGTGAC	NM_001101.3
	AS	GGAACCGCTCATTGCCAATGG	

Supplementary Table S2. Genes evaluated in the quantitative PCR array

Position	Accession No.	Symbol	Description
A1	NM_001146	ANGPT1	Angiopoietin 1
A2	NM_000038	APC	Adenomatous polyposis coli
A3	NM_004674	ASH2L	Ash2 (absent, small, or homeotic)-like (Drosophila)
A4	NM_013314	BLNK	B-cell linker
A5	NM_001755	CBFB	Core-binding factor, beta subunit
A6	NM_001295	CCR1	Chemokine (C-C motif) receptor 1
A7	NM_000591	CD14	CD14 molecule
A8	NM_001770	CD19	CD19 molecule
A9	NM_006016	CD164	CD164 molecule, sialomucin
A10	NM_001766	CD1D	CD1d molecule
A11	NM_001767	CD2	CD2 molecule
A12	NM_001242	CD27	CD27 molecule
B1	NM_001773	CD34	CD34 molecule
B2	NM_001775	CD38	CD38 molecule
B3	NM_000732	CD3D	CD3d molecule, delta (CD3-TCR complex)
B4	NM_000073	CD3G	CD3g molecule, gamma (CD3-TCR complex)
B5	NM_000616	CD4	CD4 molecule
B6	NM_000610	CD44	CD44 molecule (Indian blood group)
B7	NM_203330	CD59	CD59 molecule, complement regulatory protein
B8	NM_005191	CD80	CD80 molecule
B9	NM_006889	CD86	CD86 molecule
B10	NM_001768	CD8A	CD8a molecule
B11	NM_001805	CEBPE	CCAAT/enhancer binding protein (C/EBP), epsilon
B12	NM_001806	CEBPG	CCAAT/enhancer binding protein (C/EBP), gamma

C1	NM_015892	CHST15	Carbohydrate (N-acetylgalactosamine 4-sulfate 6-O) sulfotransferase 15
C2	NM_000757	CSF1	Colony stimulating factor 1 (macrophage)
C3	NM_000758	CSF2	Colony stimulating factor 2 (granulocyte-macrophage)
C4	NM_003467	CXCR4	Chemokine (C-X-C motif) receptor 4
C5	NM_005618	DLL1	Delta-like 1 (Drosophila)
C6	NM_000118	ENG	Endoglin (Osler-Rendu-Weber syndrome 1)
C7	NM_000799	EPO	Erythropoietin
C8	NM_005238	ETS1	V-ets erythroblastosis virus E26 oncogene homolog 1 (avian)
C9	NM_001987	ETV6	Ets variant 6
C10	NM_001459	FLT3LG	Fms-related tyrosine kinase 3 ligand
C11	NM_002049	GATA1	GATA binding protein 1 (globin transcription factor 1)
C12	NM_032638	GATA2	GATA binding protein 2
D1	NM_006037	HDAC4	Histone deacetylase 4
D2	NM_005474	HDAC5	Histone deacetylase 5
D3	NM_001098416	HDAC7	Histone deacetylase 7
D4	NM_178425	HDAC9	Histone deacetylase 9
D5	NM_000572	IL10	Interleukin 10
D6	NM_000641	IL11	Interleukin 11
D7	NM_002187	IL12B	Interleukin 12B (natural killer cell stimulatory factor 2, cytotoxic lymphocyte maturation factor 2, p40)
D8	NM_000575	IL1A	Interleukin 1, alpha
D9	NM_000586	IL2	Interleukin 2

D10	NM_018724	IL20	Interleukin 20
D11	NM_002184	IL6ST	Interleukin 6 signal transducer
D12	NM_002191	INHA	Inhibin, alpha
E1	NM_002192	INHBA	Inhibin, beta A
E2	NM_000210	ITGA6	Integrin, alpha 6
E3	NM_000214	JAG1	Jagged 1
E4	NM_002226	JAG2	Jagged 2
E5	NM_002253	KDR	Kinase insert domain receptor (a type III receptor tyrosine kinase)
E6	NM_003994	KITLG	KIT ligand
E7	NM_005574	LMO2	LIM domain only 2 (rhombotin-like 1)
E8	NM_006152	LRMP	Lymphoid-restricted membrane protein
E9	NM_002371	MAL	Mal, T-cell differentiation protein
E10	NM_007181	MAP4K1	Mitogen-activated protein kinase kinase kinase kinase 1
E11	NM_004994	MMP9	Matrix metallopeptidase 9 (gelatinase B, 92kDa gelatinase, 92kDa type IV collagenase)
E12	NM_014071	NCOA6	Nuclear receptor coactivator 6
F1	NM_000625	NOS2	Nitric oxide synthase 2, inducible
F2	NM_017617	NOTCH1	Notch 1
F3	NM_024408	NOTCH2	Notch 2
F4	NM_004557	NOTCH4	Notch 4
F5	NM_000442	PECAM1	Platelet/endothelial cell adhesion molecule
F6	NM_006017	PROM1	Prominin 1
F7	NM_002838	PTPRC	Protein tyrosine phosphatase, receptor type, C

F8	NM_005349	RBPJ	Recombination signal binding protein for immunoglobulin kappa J region
F9	NM_001754	RUNX1	Runt-related transcription factor 1
F10	NM_022754	SFXN1	Sideroflexin 1
F11	NM_144949	SOCS5	Suppressor of cytokine signaling 5
F12	NM_000582	SPP1	Secreted phosphoprotein 1
G1	NM_007315	STAT1	Signal transducer and activator of transcription 1, 91 kDa
G2	NM_003150	STAT3	Signal transducer and activator of transcription 3 (acute-phase response factor)
G3	NM_020860	STIM2	Stromal interaction molecule 2
G4	NM_003189	TAL1	T-cell acute lymphocytic leukemia 1
G5	NM_000459	TEK	TEK tyrosine kinase, endothelial
G6	NM_003265	TLR3	Toll-like receptor 3
G7	NM_138554	TLR4	Toll-like receptor 4
G8	NM_006288	THY1	Thy-1 cell surface antigen
G9	NM_003701	TNFSF11	Tumor necrosis factor (ligand) superfamily, member 11
G10	NM_006778	TRIM10	Tripartite motif containing 10
G11	NM_005428	VAV1	Vav 1 guanine nucleotide exchange factor
G12	NM_003376	VEGFA	Vascular endothelial growth factor A
H1	NM_000190	HMBS	Hydroxymethylbilane synthase
H2	NM_004048	B2M	Beta-2-microglobulin
H3	NM_000194	HPRT1	Hypoxanthine phosphoribosyltransferase 1
H4	NM_002046	GAPDH	Glyceraldehyde-3-phosphate dehydrogenase
H5	NM_000291	PGK1	Phosphoglycerate kinase 1
